# Enhancing Effect of eHealth Use on the Associations Between Social Supports and Well-Being in Japanese Employed Women Providing Childcare or Care: Bayesian Structural Equation Modeling Study

**DOI:** 10.2196/68119

**Published:** 2025-06-26

**Authors:** Noyuri Yamaji, Daisuke Yoneoka, Daichi Suzuki, Kiriko Sasayama, Erika Ota, Etsuko Nishimura, Hisateru Tachimori, Eiko Saito

**Affiliations:** 1 Institute of Clinical Epidemiology Showa Medical University Tokyo Japan; 2 Global Health Nursing Graduate School of Nursing St. Luke's International University Tokyo Japan; 3 Department of Family Nursing Division of Health Sciences and Nursing, Graduate School of Medicine The University of Tokyo Tokyo Japan; 4 Department of Epidemiology Japan Institute for Health Security Tokyo Japan; 5 Institute for Global Health Policy Research Bureau of Global Health Cooperation Japan Institute for Health Security Tokyo Japan; 6 Sustainable Society Design Center Graduate School of Frontier Sciences The University of Tokyo Chiba Japan; 7 Faculty of Nursing Komazawa Women’s University Tokyo Japan; 8 Department of Health Policy and Management Keio University School of Medicine Tokyo Japan

**Keywords:** eHealth, social support, well-being, women’s health, caregivers, working women

## Abstract

**Background:**

The increasing prevalence of information and communication technologies has made health-related information and social support more accessible on the web. However, limited evidence exists on how eHealth and social support affect the well-being of employed women who also serve as caregivers in Japan.

**Objective:**

This study aimed to assess the relationship between social support and well-being among employed Japanese women providing childcare or caregiving and explore eHealth use’s role in enhancing this relationship.

**Methods:**

We conducted a cross-sectional study using secondary data analysis from a nationwide web-based questionnaire survey of 10,000 employed women aged 20-65 years, administered from February 28, 2023, to March 7, 2023. The primary study used a quota random sampling approach based on age and geographic area from the research company’s panel. For this analysis, we focused on a subgroup of 2456 women who reported either caring for children less than 7 years old or providing other caregiving responsibilities. We employed a Bayesian structural equation model to estimate the enhancing effect of eHealth on the relationship between social support and 4 well-being indicators: life satisfaction, worthwhileness, happiness, and anxiety.

**Results:**

Among the 2456 employed women included, 1784 (72.6%) received social support and 1635 (66.6%) obtained health-related information via eHealth. Bayesian structural equation model analysis revealed that the standardized total effects of social support on well-being were 0.20 (95% CI 0.13-0.27) in the group without eHealth use and 0.47 (95% CI 0.45-0.50) in the group with eHealth use.

**Conclusions:**

The findings suggest that eHealth may enhance the positive impact of social support on the well-being of employed Japanese women providing childcare or caregiving. This study highlights the potential of eHealth interventions in supporting social support and well-being among working women with caregiving responsibilities in Japan.

## Introduction

Well-being is defined as a multifaceted concept encompassing happiness, satisfaction, personal growth, fulfillment, and contribution to the community [1]. It includes physical, emotional, and social dimensions and is a key factor in determining women’s productivity and quality of life. While employment has been shown to improve women’s well-being [2], it also exposes them to adverse effects from work-family conflict [3]. Women in the prime of their working lives face various health challenges, including mental health issues, work-related illnesses, and reproductive health concerns [4]. Working women with childcare or older adult care responsibilities must balance their work and home lives, and this double burden affects their well-being [5,6].

Social support, a key factor in maintaining well-being, traditionally comes from family, friends, and the community [7]. Social support from family and friends plays a pivotal role in maintaining physical and mental health. This multifaceted concept revolves around interpersonal interactions, wherein individuals perceive reliable connections with friends or family members on whom they can depend during favorable and challenging circumstances [8]. Good social relationships provide emotional and practical resources essential for feeling nurtured and esteemed, consequently fostering healthier lifestyles [9]. Social support has protective physical and mental effects [10]. Although social support from partners is essential [11], approximately 90% of households with children in Japan are nuclear families [12]. The traditional sources of social support may be limited for many working women. This situation creates a need for alternative forms of support, particularly for those balancing work with childcare or older adult care responsibilities.

The rapid digitalization of Japanese society has also opened new avenues for addressing this need. In Japan, with over 90% of the working-age population using the internet and about 80% engaging with social networking services [13], eHealth has emerged as a promising solution. eHealth refers to the cost-effective and secure use of information and communication technologies (ICT) to support health and health-related fields [14]. The dissemination of health information via the internet has expanded in recent years and information on social support has also increased [15]. Recent trends indicate that social support is being increasingly accessed and provided on the web through various digital platforms and social networks. For example, several interventions have been developed that incorporate social support content using group phone calls or applications [16,17].

This shift reflects a broader societal change toward digital communication, which enables individuals to access information and community and emotional support without geographical constraints [18]. Internet-based social support has shown potential in enhancing well-being by providing immediate access to resources and reducing social isolation [19]. It is also known as an important form of social support. These eHealth tools may provide crucial social support and be valuable for working women facing time constraints and the social stigma associated with seeking help. However, these tools are primarily designed for specific groups, such as pregnant women or women with health issues. Few studies specifically targeting working women exist.

In addition, cultural differences must be considered when implementing eHealth interventions. Cultural perspectives on the self differ between Western and Eastern societies. In Western cultures, there is a tendency to view individuals as autonomous entities. Conversely, Eastern cultures often conceptualize the self as intrinsically interconnected with others, emphasizing social relationships and collective harmony [20]. This fundamental difference in self-perception influences various aspects of social behavior. A previous study found cultural differences in the emotional correlates of social support receipt across countries, highlighting the importance of considering cultural context when examining social support interventions. In addition, seeking social support might help the Japanese reduce loneliness [21].

Given Japan’s unique cultural and societal context, including its high technological adoption rate, it is necessary to understand how social support and eHealth use interact to influence the well-being of working women with caregiving responsibilities. However, there have been limited studies on the relationship between social support and well-being among employed women raising children or caring for them and the relationship between the use of eHealth medical information in Japan. Therefore, this study aimed to fill this gap by evaluating the relationship between social support and well-being among employed women providing childcare or caregiving and the enhancing effect of eHealth use on this relationship. We hypothesized that individuals who use eHealth services will have improved well-being because they receive support from internet-based communities in addition to support from close family and friends. In this study, we hypothesized that the enhancing variable, “eHealth use,” would influence the direct effect of “social support” on “well-being.”

## Methods

### Study Design

**Figure 1:**
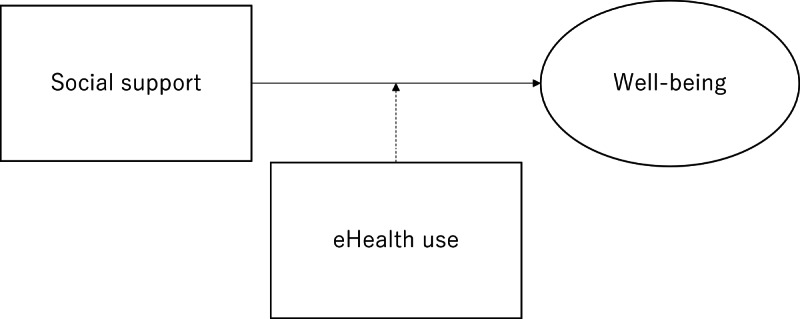
Hypothetical model.

This is a cross-sectional study using secondary data from a web-based nationwide questionnaire survey conducted from February 28, 2023, to March 7, 2023 [22]. Figure 1 illustrates the hypothetical model of the Bayesian structural equation model (BSEM).

### Participants and Data

The data used in this study were obtained from a nationwide survey of 10,000 employed women aged 20-65 years in Japan using a questionnaire developed for the previous study [22]. This primary study included participants who met the following criteria: (1) being 20-65 years old, (2) being employed, and (3) being able to provide informed consent. Students were excluded. A research company conducted recruitment and data collection using a web-based questionnaire. This company used its extensive nationwide panel, which includes women from diverse backgrounds across all 47 prefectures of Japan. A link containing the study introduction and the questionnaire was sent to the company’s research panel. We stratified the age categories and prefectures for recruitment to improve sample diversity. A questionnaire was distributed using the web-based platform of a professional research company. The first page of the questionnaire contained an informed consent form and the participants could access the questionnaire by checking the agreement button at the end of the page. A structured questionnaire contained questions about demographics (eg, age, sex, and comorbidities), socioeconomic status, social support, health status, well-being, and eHealth. All monetary values originally reported in Japanese yen were converted to US dollars using the average exchange rate at the time of data collection, which was 1 JPY=0.0069 USD. The participants required approximately 10-15 minutes to complete the entire questionnaire. Eligible participants could then choose to respond to the questionnaire.

From the initial 10,000 respondents, participants for this study were selected based on the following criteria: women raising or caring for children aged <7 years were included in the analysis. Those who met these criteria were included in the final analysis.

### Measures and Outcomes

#### Well-Being

The Office for National Statistics (ONS)-4 questionnaire, developed by the United Kingdom Office for National Statistics, was used to measure personal well-being [23]. This questionnaire consists of 4 items: life satisfaction, worthwhileness, happiness, and feelings of anxiety. The participants were instructed to rate their well-being on a scale of 0-10. For life satisfaction, feeling that things done in life are worthwhile and happiness scores are categorized as low (0-4), medium (5-6), high (7-8), and very high (9-10). Feelings of anxiety were categorized as very low (0-1), low (2-3), medium (4-5), or high (6-10). The scores were analyzed using these threshold categories [23].

#### Social Support

The participants were asked multiple-choice questions about the people or groups who supported them in child and nursing care, which we calculated using dichotomous data: yes or no support.

#### Usage of eHealth

In this study, eHealth use was defined as obtaining health-related information through ICT. The participants were asked how they obtained health-related information. The choices included medical professionals, health events, books (newspapers, scientific journals, and scientific papers), television, internet news, internet search engines, social network services, medical information sites, blogs, information from the government and local government, family, friends, and others. If the participants selected ICT-enabled health care services from the options (eg, internet news, internet search engines, social network services, medical information sites, and blogs), we recorded them as dichotomous data for eHealth use (yes or no).

### Statistical Analysis

**Figure 2:**
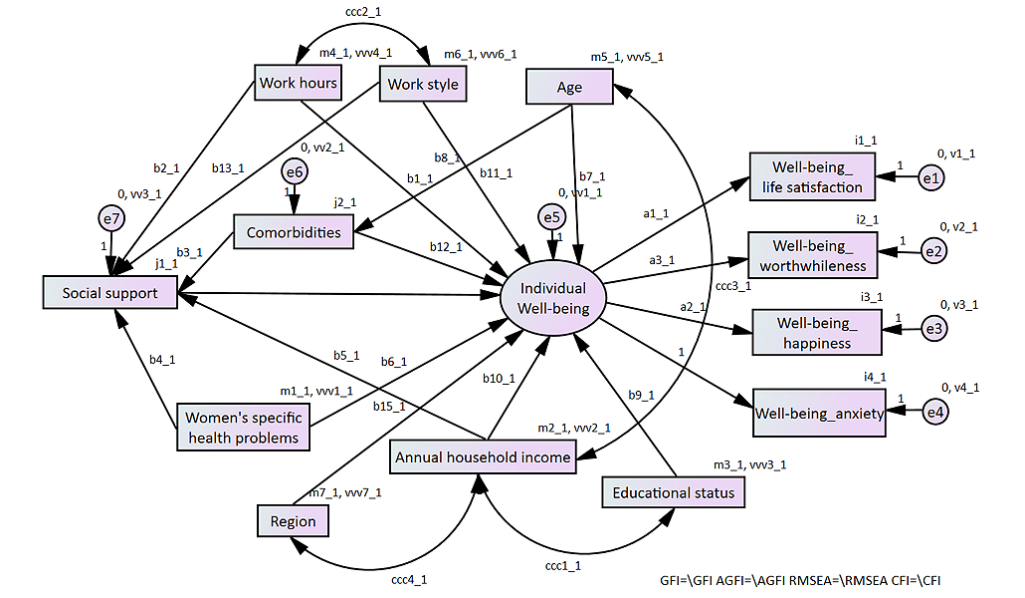
Preidentified structural model.

First, a descriptive analysis was performed to examine participant background characteristics and well-being. The Kendall rank correlation coefficient was used to confirm the latent variables that should be considered in addition to the hypothetical model. We selected confounders shown in previous studies to be associated with well-being and social support [24-30], including age, region, educational status, annual household income, work style, work hours, comorbidities, and specific health problems (Figure 2). A BSEM was used to assess the enhancing effect of eHealth usage on the relationship between social support and the 4 well-being items. We adopted the BSEM approach because it accommodates smaller sample sizes [31] and allows for a natural and explicit representation of Likert scales and binary latent variables [32,33]. The BSEM assumes one latent variable encompassing 4 well-being items and their correlated residuals (Figure 2). Based on the structure, we grouped the participants using eHealth and presented the results separately. We estimated the parameters in the BSEM for the groups with and without eHealth use, respectively. In addition, the ONS-4 data are ordinal scale, and treating them as normally distributed metric values is considered inappropriate. Therefore, we analyzed them using an ordered probit model, assuming the latent variable follows a standard normal distribution, which is appropriate for modeling binary outcomes. The prior distribution was set as a standard normal distribution, reflecting the assumption that the coefficients are centered around zero. We estimated the total standardized effects as path coefficients and their 95% CIs. The standardized total effect normalized the total effect by considering the ratio of the SDs of social support to well-being. The posterior distribution was derived using the Markov Chain Monte Carlo algorithm. The path coefficient was considered significant if the 95% CI excluded zero. The maximum number of iterations was set to 100,000 with 500 burn-in iterations. We judged that the algorithm converged after 100,000 sampling iterations, as determined by a convergence statistic below the threshold of 1.002 [34]. The estimated model was evaluated by the deviance information criterion. Finally, we performed the sensitivity analysis concerning model specifications involved in testing alternative model structures by omitting paths and latent variables to ensure the robustness of the results. Specifically, we confirmed that the standardized coefficients among well-being, eHealth, and other variables did not significantly change by omitting region and work hours and the paths that were linked to them, respectively. All statistical analyses were performed using the SPSS Statistics (version 29.0.1; IBM Corp) software and SPSS Amos (version 29.0; IBM Corp), and a *P* value of <.05 was considered statistically significant.

### Ethical Considerations

This study was approved by the Research Ethics Committee of St. Luke’s International University (reference number 22-A089). All participants provided informed consent on the web and the study data were collected anonymously. The research company offered the participants shopping points as rewards.

## Results

### Demographic Characteristics

Table 1 presents the demographic characteristics of the 2456 women who met the inclusion criteria. The table includes information on age, occupation, region, educational status, annual household income, work style, daily working hours, comorbidities, and women’s specific health problems.

**Table 1:** Demographic characteristics (N=2456).

Variable and classification			Values		eHealth use				*P* value
					With (n=1635)		Without (n=821)		
Age (years), mean (SD)			38.3 (8.9)		37.7 (9.2)		38.6 (8.8)		
**Age (years), n (%)**									.09
	20-30	479 (19.5)		293 (17.9)		186 (22.7)			
	30-40	844 (34.4)		572 (35.0)		272 (33.1)			
	40-50	860 (35.0)		581 (35.5)		279 (34.0)			
	50-60	209 (8.5)		144 (8.8)		65 (7.9)			
	60-65	64 (2.6)		45 (2.8)		19 (2.3)			
**Occupation, n (%)**									.18
	Management or executive	26 (1.1)		18 (1.1)		8 (1.0)			
	Company employee (full-time)	979 (39.9)		662 (40.5)		317 (38.6)			
	Company employee	111 (4.5)		67 (4.1)		44 (5.4)			
	Company employee (temporary employee)	80 (3.3)		58 (3.5)		22 (2.7)			
	Public employee (excluding teaching staff)	87 (3.5)		54 (3.3)		33 (4.0)			
	Faculty member	89 (3.6)		60 (3.7)		29 (3.5)			
	Health care provider	209 (8.5)		123 (7.5)		86 (10.5)			
	Professional	7 (0.3)		5 (0.3)		2 (0.2)			
	Independent business or self-employed workforce	109 (4.4)		80 (4.9)		29 (3.5)			
	Part-time work	759 (30.9)		508 (31.1)		251 (30.6)			
**Region, n (%)**									.30
	Hokkaido, Tohoku	342 (13.9)		213 (13.0)		129 (15.7)			
	Kanto	879 (35.8)		605 (37.0)		274 (33.4)			
	Tokai	326 (13.3)		44 (2.7)		24 (2.9)			
	Hokuriku	68 (2.8)		205 (12.5)		121 (14.7)			
	Kinki	421 (17.1)		281 (17.2)		140 (17.1)			
	Chugoku	137 (5.6)		95 (5.8)		42 (5.1)			
	Shikoku	62 (2.5)		45 (2.8)		17 (2.1)			
	Kyuusyu, Okinawa	221 (9.0)		147 (9.0)		74 (9.0)			
**Educational status, n (%)**									.11
	Elementary school	34 (1.4)		24 (1.5)		10 (1.2)			
	High school	494 (20.1)		314 (19.2)		180 (21.9)			
	Associate degree	340 (13.8)		241 (14.7)		99 (12.1)			
	Diploma	471 (19.2)		297 (18.2)		174 (21.2)			
	University bachelor	1024 (41.7)		693 (42.4)		331 (40.3)			
	MSc or PhD	93 (3.8)		66 (4.0)		27 (3.3)			
**Annual household income^a^, n (%)**									.01
	<2,000,000 JPY (US $13,800)	171 (7.0)		98 (6.0)		73 (8.9)			
	2,000,000-4,000,000 JPY (US $13,800-27,600)	406 (16.5)		263 (16.1)		143 (17.4)			
	4,000,000-6,000,000 JPY (US $27,600-41,400)	604 (24.6)		415 (25.4)		189 (23.0)			
	6,000,000-8,000,000 JPY (US $41,400-55,200)	513 (20.9)		329 (20.1)		184 (22.4)			
	8,000,000-10,000,000 JPY (US $55,200-69,000)	389 (15.8)		257 (15.7)		132 (16.1)			
	≥10,000,000 JPY (US $69,000)	372 (15.1)		272 (16.6)		100 (12.2)			
	Missing	1 (—^b^)		—		—			
**Work style, n (%)**									.02
	Day shift	2252 (91.7)		1513 (92.5)		739 (90.0)			
	Night shift	204 (8.3)		122 (7.5)		82 (10.0)			
**Daily working hours** **, n (%)**									.40
	<8	1908 (77.7)		1262 (77.2)		646 (78.7)			
	≥8	548 (22.3)		373 (22.8)		175 (21.3)			
**Comorbidities^c^, n (%)**									.06
	Without	1934 (78.8)		1269 (77.7)		665 (81.0)			
	With	521 (21.2)		365 (22.3)		156 (19.0)			
**Women’s specific health problems^d^, n (%)**									<.01
	Without	835 (34.0)		437 (26.7)		398 (48.5)			
	With	1620 (66.0)		1197 (73.3)		423 (51.5)			
	Missing	1 (—)		—		—			

^a^As of May 2025, a currency exchange rate of 1 JPY=US $0.0069 was applicable.

^b^Not available.

^c^Comorbidities include hypertension, heart disease, atherosclerotic disease, diabetes mellitus, hyperlipidemia, and chronic kidney disease.

^d^Women’s specific health problems include menstrual symptoms and illnesses, premenstrual syndrome, cancers common in women (eg, cervical, uterine, ovarian, and breast cancer), pregnancy- and childbirth-related symptoms, menopausal disorders, mental disorders, insomnia, infertility, endometriosis, benign female tumors, blood flow disorders, anemia, gastrointestinal disorders, headaches and migraines, underweight status, nutritional disorders, autoimmune diseases, and pelvic floor symptoms and diseases.

Approximately 70% of the participants were in their 30s and 40s. About 40% of participants were employed by a company and worked full-time. Most participants lived in the Kanto region, followed by Kinki, with the fewest residing in Tokai and Shikoku. Approximately half of the participants had graduated from graduate school or university. Most participants reported middle-class household incomes, with the most common range being upper-middle income (4-6 million yen [US $27,600-41,400] annually). This was followed by households with middle-high income (6-8 million yen [US $41,400–55,200]) and lower-middle income (2-4 million yen [US$ 13,800-27,600]). A notable portion, 372 (15.1%) participants, belonged to high-income households, earning over 10 million yen (over US $69,000) annually. The Kanto region had a higher concentration of high-income households, with 194/879 (22.1%) of participants from this area reporting annual incomes over 10 million yen (over US $69,000). Approximately 204/2456 (8.3%) participants worked night shifts, and 548/2456 (22.3%) participants worked an average of ≥8 hours per day. A total of 521 out of 2456 (21.2%) participants had comorbidities. More than half of the participants (1620/2456, 66% women) answered that they were experiencing symptoms that are common in women, such as premenstrual syndrome, female-specific cancers (uterine cancer and breast cancer), pregnancy-related issues, menopausal disorders, and mental disorders. Of the 2456 participants, 2123 (86.4%) had obtained health-related information in some form, including through means other than the internet, and 1635 out of 2456 (66.6%) had obtained health-related information through eHealth. The breakdown of eHealth sources was as follows: search engines (1003/2456, 40.8%), internet (851/2456, 34.6%), social networking service (744/2456, 30.3%), medical information sites (137/2456, 5.6%), and blogs (126/2456, 5.1%).

### Profiles and Correlations of Measures

**Figure 3:**
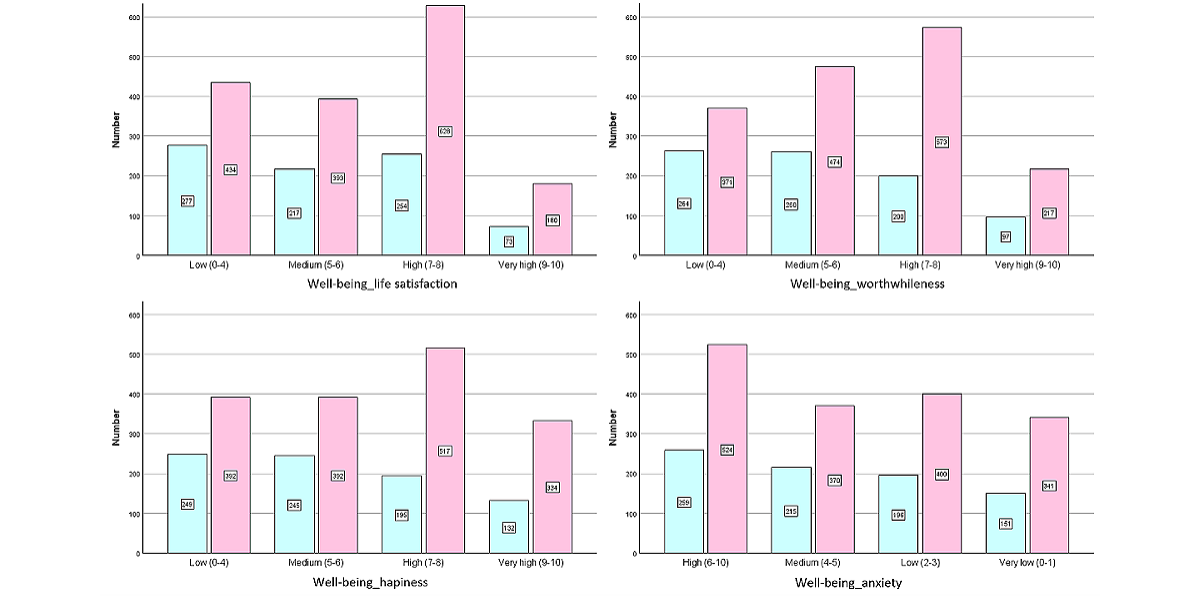
Well-being classification by presence or absence of eHealth.

In terms of well-being, 711 out of 2456 (28.9%) participants reported low life satisfaction, 635 out of 2456 (25.9%) felt their lives were less worthwhile, and 641 out of 2456 (26.1%) had low happiness. Conversely, 253 out of 2456 (10.3%), 314 out of 2456 (12.8%), and 466 out of 2456 (19.0%) participants reported high scores for life satisfaction, feeling worthwhile, and happiness, respectively. In addition, 783 out of 2456 (31.9%) participants experienced high anxiety, whereas 492 out of 2456 (20.0%) reported low anxiety (Figure 3). Social support was reported by 1784 out of 2456 (72.6%) women. Table 2 presents the correlation coefficients between eHealth use, social support, and well-being measures. Significant correlations were found between well-being, social support, and eHealth use, although the effect size was small (Table 2).

**Table 2:** Correlation matrix (N=2456).

Variables								Values		1		2		3		4		5		6		
	**eHealth use**																					
			n (%)				1635 (66.6)															
				Correlation, tau_b						—^a^												
				*P* value						—												
	**Social support**																					
					n (%)			1784 (72.6)														
					Correlation, tau_b					.165		—										
					*P* value					<.01		—										
**Well-being**																						
			**Life**																			
						Mean (SD)	2.28 (0.99)															
						Correlation, tau_b			.084		.127		—									
						*P* value			<.01		<.01		—									
			**Value**																			
						Mean (SD)	2.31 (0.99)															
						Correlation, tau_b			.105		.123		.715		—							
						*P* value			<.01		<.01		<.01		—							
			**Happiness**																			
						Mean (SD)	2.40 (1.07)															
						Correlation, tau_b			.094		.132		.678		.658		—					
						*P* value			<.01		<.01		<.01		<.01		—					
			**Anxiety**																			
						Mean (SD)	2.32 (1.12)															
						Correlation, tau_b			.02		.112		.188		.152		.196		—			
						*P* value			.296		<.01		<.01		<.01		<.01		—			

^a^Not available.

### Enhancing Effects of eHealth Use

The direct effects of associations between well-being and social support, as BSEM, differed depending on eHealth use. Among participants without eHealth use, the standardized total effects were 0.20 (95% CI 0.13-0.27, SE 0.00, SD 0.03), with a convergence diagnostic value of 1.00. In contrast, for those with eHealth use, the standardized total effect was higher at 0.47 (95% CI 0.45-0.50, SE 0.00, SD 0.01), with the same convergence diagnostic value of 1.00. The posterior predictive distribution *P* value was .50 and the deviance information criterion was 19,262.24, suggesting strong predictive validity of the model. The sensitivity analysis showed that the key relationships in the original model were robust to these changes as the estimated path coefficients and factor loadings varied only marginally. This result suggests that the model’s conclusions are not dependent on specific assumptions regarding the model structure.

## Discussion

### Summary of Study Findings

This study examined the characteristics of participants and the relationship among well-being, social support, and eHealth use among employed women providing childcare or care. In this study, eHealth use enhanced the relationship between social support and well-being, with higher standardized total effects observed in the group that used eHealth compared to those that did not. Our findings align with recent research on eHealth interventions, demonstrating their positive impact on social support and well-being. A study suggested that social media use was positively associated with perceived responsiveness in internet-based social networks, which improved perceived social support and was linked to reduced loneliness and increased life satisfaction [19]. Furthermore, DeHoff et al [35] showed that internet-based social support might enhance participants’ belief in being supported, leading to improved health behaviors. These findings suggest that eHealth use may contribute to improved well-being by facilitating access to social support and enhancing individuals’ perception of being supported. It might be particularly effective for working women who have limited time due to childcare or caregiving responsibilities.

Social support refers not only to the receipt of support but also to an individual’s perception of support from family, friends, and significant others in their lives [36,37]. Current research suggests that family and close friends are positively associated with emotional well-being, even when they are far away [38]. Furthermore, recent research has shown that internet-based resources allow individuals to connect with and receive social support from people other than family and close friends [18]. The results of this study also support this hypothesis, as internet-based connections with individuals or organizations may lead to social support and improve individual well-being. A study investigating internet-based social support for parents of children with medical needs also showed that, through internet-based support, they receive support from peers, learn the information they need, and acquire the ability to deal with the associated emotional challenges [35]. As nuclear families make up the majority of households in Japan [12], women involved in the care of children and older family members need to be supported effectively using the internet as a tool for exchanging information about these fields and creating social connections for support. For Japanese people, who place great importance on social relationships and group harmony, this intervention might enhance their support-seeking behavior and reduce loneliness [21].

Previous research has demonstrated that cognitive factors such as age [39], education level, income [40], gender, occupational status, and marital status influence eHealth literacy [41]. In addition, a previous study indicated that higher health literacy levels positively influence health-seeking attitudes and social support [42]. Our study found minimal differences between groups based on eHealth usage. This result may be attributed to the data collection method, which was an internet-based survey, and our focused sample of employed Japanese women aged 20-65 years engaged in childcare or caregiving activities [43]. Most participants were in their 30s and 40s, with approximately half having completed higher education. These individuals are not older adults, have a higher level of education, and are employed women. Therefore, they are likely to belong to a group with a relatively high rate of eHealth usage. Regarding income, the participants in this study were employed women whose annual household income exceeded the average income in Japan (5,242,000 yen [US $36,368.68]). This is likely because the survey was conducted among working women, many of whom belong to dual-income households. The lack of comparative data makes it difficult to make a general statement. As many studies have shown a relationship between annual household income and well-being [44,45], the participants in this study may represent a group with a relatively high level of well-being. Health-related conditions, such as high blood pressure and female-specific conditions, were prevalent among the participants. Other well-being surveys in the United Kingdom have shown that the well-being of Japanese people tends to be relatively low [46]. Compared with the British survey using ONS-4, participants had a lower life satisfaction rate and a higher percentage of individuals with high anxiety [46]. As a previous study has pointed out, social support interventions should account for cultural context and cognitive factors and be developed and implemented as tailored eHealth interventions for a similar population.

In addition, when implementing eHealth, factors that might influence its use should be considered. Previous studies have not reached a consensus on the association between eHealth service use, including social media, and well-being. Although some studies have reported a positive association with well-being [36,47], others have suggested that social media use negatively affects well-being [48,49]. Given the complex factors influencing eHealth literacy and well-being, it is important to consider how eHealth service use may impact well-being outcomes across different populations [50]. Previous studies have suggested that interactive methods may have positive effects and that internet-based information exchange and positive social interaction enhance perceived social support and well-being [49]. However, we could not clarify the specific methods for obtaining information via eHealth in this study. Future studies should investigate how individuals obtain information through eHealth services.

### Implications for Future Practice and Research

These findings suggest that eHealth use can play a significant role in enhancing well-being among women, particularly those providing childcare or caring for someone else. Labor participation by women has increased globally, benefiting individuals and society worldwide [51]. Japan is no exception, with the number increasing by more than 4 million over the past decade [52]. However, gender inequality exists in Japan’s working environment. In 2023, the World Economic Forum assessed the state and evolution of the gender gap across 4 key dimensions (economic participation and opportunity, educational attainment, health and survival, and political empowerment). The World Economic Forum ranks Japan 125th among 146 countries [53]. Various issues were identified, including the gender pay gap, a lack of female executives, and a decline in the number of women in regular employment after their 30s [54]. Women’s progress in social advancement is closely linked to changes in daily life and overall well-being. Using internet-based social support to enhance women’s well-being could promote their social advancement, improve the next generation’s health, and address gender equality. This finding suggests that health care providers and policy makers should consider promoting eHealth platforms to improve mental and physical health outcomes. Moreover, they must support women in accessing required information via eHealth services. Future studies can further explore the mechanisms through which eHealth influences social support and well-being, potentially focusing on the most effective eHealth interventions or platforms. eHealth interventions should be developed to address the specific needs of employed women providing childcare or caregiving and to assess their benefits and harms. In addition, longitudinal studies can provide insights into the long-term effects of eHealth use on well-being and other health outcomes.

### Strengths and Limitations

This study is unique in that it examined the relationship among well-being, social support, and eHealth use among a large sample of working women raising or caring for children. The use of BSEM allowed for a robust analysis of the data, providing CIs and a sensitivity analysis that strengthened the validity of the results. Focus on the use of eHealth provides valuable insights into an emerging area of health intervention that is particularly relevant in today’s digital age. However, this study has some limitations. First, the cross-sectional design restricted the ability to infer causality between eHealth use and well-being. Second, our study did not use validated scales specifically designed to measure social support, work-life balance, job satisfaction, and caregiver burden, which could have provided more robust and standardized assessments of these crucial variables. In addition, we did not investigate perceived social support in this study. However, it is important to consider that the mechanisms linking social support and well-being are complex and involve multiple correlated factors. Including perceptions of social support may provide a more comprehensive understanding of how these elements interact and contribute to overall well-being. Third, we only assessed the use of ICT to obtain health-related information, such as the usage of eHealth, and could not evaluate social support through ICT. The frequency or intensity of use was also not evaluated. As eHealth encompasses a broader range of concepts, further studies should include this in evaluating ICT for support in health and health-related fields. Fourth, these data were collected through a self-reported questionnaire, and response biases may be present.

### Conclusion

This study suggests that using eHealth services for employed women raising children or caring for someone may enhance the impact of social support on their well-being. However, the complexity of the relationship between eHealth use, social support, and well-being necessitates further investigation. Further studies should investigate the following points while considering cultural contexts and individual differences in eHealth literacy: the mechanisms underlying the relationship among social support, well-being, and eHealth use; the potential benefits and harms of eHealth interventions; and the long-term effects of eHealth interventions on social support and well-being. In addition, further research is needed to develop customized eHealth interventions that address the specific needs of working women with caregiving responsibilities. Deepening our understanding in these areas would allow for the implementation of more effective eHealth interventions, contributing to the improvement of working women’s well-being.

## Data Availability

The data used in this study are not publicly available because of privacy and confidentiality concerns. Consequently, external parties cannot share the data.

## Abbreviations

BSEM: Bayesian structural equation model
ICT: information and communication technologies
ONS: Office for National Statistics
